# Depth-resolved assessment of changes in concentration of chromophores using time-resolved near-infrared spectroscopy: estimation of cytochrome-c-oxidase uncertainty by Monte Carlo simulations

**DOI:** 10.1364/BOE.10.004621

**Published:** 2019-08-16

**Authors:** Aleh Sudakou, Stanislaw Wojtkiewicz, Frédéric Lange, Anna Gerega, Piotr Sawosz, Ilias Tachtsidis, Adam Liebert

**Affiliations:** 1 Nalecz Institute of Biocybernetics and Biomedical Engineering Polish Academy of Sciences, Trojdena 4, 02-109 Warsaw, Poland; 2 Department of Medical Physics and Biomedical Engineering, University College London, London WC1E 6BT, United Kingdom; 3 School of Computer Science, University of Birmingham, Edgbaston, Birmingham, B15 2TT, United Kingdom

## Abstract

Time-resolved near-infrared spectroscopy (TR-NIRS) measurements can be used to recover changes in concentrations of tissue constituents (ΔC) by applying the moments method and the Beer-Lambert law. In this work we carried out the error propagation analysis allowing to calculate the standard deviations of uncertainty in estimation of the ΔC. Here, we show the process of choosing wavelengths for the evaluation of hemodynamic (oxy-, deoxyhemoglobin) and metabolic (cytochrome-c-oxidase (CCO)) responses within the brain tissue as measured with an in-house developed TR-NIRS multi-wavelength system, which measures at 16 consecutive wavelengths separated by 12.5 nm and placed between 650 and 950 nm. Data generated with Monte Carlo simulations on three-layered model (scalp, skull, brain) for wavelengths range from 650 to 950 nm were used to carry out the error propagation analysis for varying choices of wavelengths. For a detector with a spectrally uniform responsivity, the minimal standard deviation of the estimated changes in CCO within the brain layer, $\sigma \Delta C_{\text{CCO}}^{\text{brain}}$ = 0.40 µM, was observed for the 16 consecutive wavelengths from 725 to 912.5 nm. For realistic a detector model, i.e. the spectral responsivity characteristic is considered, the minimum, $\sigma \Delta C_{\text{CCO}}^{\text{brain}}$ = 0.47 µM, was observed at the 16 consecutive wavelengths from 688 to 875 nm. We introduce the method of applying the error propagation analysis to data as measured with spectral TR-NIRS systems to calculate uncertainty of recovery of tissue constituents concentrations.

## 1. Introduction

Functional near-infrared spectroscopy (fNIRS) has showed capability to estimate changes in concentrations of chromophores contained in the brain: oxy-, deoxyhemoglobin and the oxidation state of cytochrome-c-oxidase (CCO) enzyme [1]. Fields of application of near-infrared spectroscopy (NIRS) method have been recently reviewed [1–5] and a common interest on using NIRS to monitor CCO emerges. CCO is present in all mitochondria and is involved in more than 95% of oxygen consumption [6]. Clinical NIRS measurements of cerebral cytochrome-c-oxidase can yield information about energy metabolism on cellular level [7] and have potential to be a metabolic marker of brain injuries [8]. The estimation of CCO is challenging and requires high accuracy in measurements as the CCO has broad spectral absorption peak in NIR region and low cerebral concentration [1]. Hence, the optical signals resulting from changes in cerebral concentration of CCO are much smaller than the signals corresponding to e.g. hemoglobins.

fNIRS relies on changes in the optical properties of tissue to evaluate physiological responses. Optical signals are measured typically in reflectance geometry, where source and detector optodes are positioned on the head surface at a distance of several centimeters (typically 3-4 cm). The changes in the optical signals can originate in the brain, in the overlying scalp or in both tissues. A reliable method to separate signals originating at different depths is required to discriminate between cerebral and systemic physiological changes [9–14] and hence avoid false-positive results in functional studies [15,16]. Number of methods have been suggested and applied to address the problem of contamination by the perfusion of extracerebral tissues when trying to recover brain oxygenation, including with the use of continuous wave sources [17] or the time-resolved NIRS (TR-NIRS) technique [18]. In TR-NIRS, short pulses of light, typically on the order of picoseconds in width, are emitted into the tissue and the arrival times of remitted photons are measured using time-correlated single photon counting electronics. The histogram of the arrival times represents the distribution of time of flight of photons (DTOF). TR data analysis methods calculate the broadening of DTOFs to recover absorption and scattering properties of tissue penetrated by the light pulses. In this study, we analyze DTOFs with the moments method [19], which utilizes statistical moments of the time-resolved distributions and allows to recover changes in the absorption coefficient with depth discrimination [20–22]. The moments method has been validated using Monte Carlo simulations [19], in experiments on phantoms [23] and during functional stimulation experiments [20,21]. The method has been applied during carotid surgery [22], to communicate with patients who are in a functionally locked-in state [24], and for assessment of cerebral perfusion by monitoring the inflow and the washout of an injected optical contrast agent indocyanine green with discrimination between the extra- and intracerebral tissue compartments [25,26]. The estimated depth-resolved changes in absorption coefficient at multiple wavelengths can be converted to depth-resolved changes in concentrations of chromophores using the Beer-Lambert law [20,22]. Literature shows lack of reported uncertainties [22] or the uncertainty is calculated as the standard deviation of block-averaged measurements [20]. Here, we extend the error propagation for the moments method [27] in order to calculate the standard deviations in the estimated changes in concentrations of chromophores within multiple layers applied for TR-NIRS data analyzed using the moments method and the Beer-Lambert law. The error propagation as used in this study accounts for the noise associated with the stochastic nature of the scattered photons, which are assumed to follow the Poisson statistics.

Here we introduce the method of applying the error propagation analysis to data as measured with spectral TR-NIRS systems to minimize uncertainty of recovery of tissue constituents’ concentrations. A recent review [3] summarizes the past studies that found the optimal number and range of wavelengths for different models, number of chromophores and number of wavelengths. The past studies aimed to minimize certain criteria, i.e. the condition number [28] and the residual norms [29] of the absorption extinction coefficients matrix, the levels of separability and cross-talk [30], the signal-to-noise ratio assuming a fixed amount of power [31], sensitivity and resolution [32], sensitivity overlap for different wavelengths [33], and a heuristic search for wavelengths that produce the closest result to the result of using 121 wavelengths [34]. Monte Carlo (MC) simulations are the gold standard for generating TR-NIRS data [35]. As such, we used MC simulated spectral TR-NIRS data for wavelengths from 650 to 950 nm for a three-layered model (scalp, skull and brain). Further, we calculated the standard deviation in estimation of concentration changes (σΔC) of oxy-, deoxyhemoglobin and CCO in two compartments (scalp, brain) for different choices of wavelengths. The wavelength optimization criteria is to minimize σΔC of CCO in the brain layer. The multi-wavelength TR-NIRS system, developed in the author’s group and successfully tested in brain hemodynamic studies [23,25], can measure DTOFs simultaneously at 16 consecutive wavelengths separated by 12.5 nm and placed between 650 nm and 950 nm. We carried out analysis within the instrument wavelength range considering two case studies: 16 consecutive wavelengths separated by 12.5 nm and varying number of evenly spread wavelengths. We included the spectral responsivity [36] of photosensitive element in the analysis. The multialkaline cathode, as used in the TR-NIRS system investigated in this paper, has much higher sensitivity at shorter wavelengths and as such the detector performance can be the dominant factor that affects the optimal wavelengths range.

## 2. Methodology

### 2.1 Error propagation in the moments method

The analysis of TR-NIRS measurements based on changes in statistical moments of DTOFs has been presented in [19,20,37]. The DTOF is a histogram of photon counts (*N_i_*) at time channels indexed by *i*. A time channel in DTOF corresponds to the time of flight of a detected photon. The multi-wavelength TR-NIRS system, as shown in [23,25], records DTOFs typically with 1024 time channels of width *t* = 13.68 picoseconds. The first three statistical moments of DTOF can be defined as: *m*_0_* = N*_tot_ *= *Σ*N_i_* (zeroth), *m*_1_ *=*  <*t*> *= *Σ*t_i_N_i_/m*_0_ (first) and $m_{2}^{c}$ = *V = *Σ(*t_i_* – *m*_1_)^2^*N_i_/m*_0_ (second central). These statistical moments of DTOF are known as: total number of photons (*N*_tot_), mean time of flight (<*t*>) and variance (*V*). The sensitivity factors relate changes in statistical moments measured for a given source-detector pair to changes in absorption coefficient Δ*μ*_a_ within sub-volumes (e.g. layers indexed by *j*): Δ*A**** ****= *-ln*(N*_tot_*/*N*_tot_*) *=* *Σ[*MPP*(*j*)Δ*μ*_a_(*j*)], Δ*T**** ***=  <*t*>*-<*t*>* *=* *Σ[*MTSF*(*j*)Δ*μ*_a_(*j*)], Δ*V* = V^*^ - *V* = Σ[*VSF*(*j*)Δ*μ*_a_(*j*)]. The star (*) denotes a statistical moment after a change in absorption, *MPP* is the mean partial pathlength, *MTSF* is the mean time of flight sensitivity factor and *VSF* is the variance sensitivity factor. The sensitivity factors can be obtained with a Monte Carlo simulation, or using analytical solutions of the diffusion approximation of light transport for simple geometries, or using the finite element method for heterogeneous models. The recovery method that uses the sensitivity factors relies on the assumptions that changes in statistical moments are linear within corresponding changes in absorption coefficient *μ*_a_±Δ*μ*_a_ which is true for Δ*μ*_a_→0. Additionally, the scattering properties should remain constant (Δ*μ*′_s_ = 0, where *μ*′_s_ is the reduced scattering coefficient). The error propagation analysis proposed in [27] allows to calculate the standard deviation in recovered absorption change (*σ*Δ*μ*_a_) within *j*–th layer for assumed heterogeneous background optical properties. *σ*Δ*μ*_a_ is calculated from the standard deviations of the three statistical moments, which may be statistically dependent as they are derived from the same DTOF curve. The existing covariances between ΔA, ΔT and ΔV are used in the error propagation model to account for a mutual statistical dependence. Assuming the dominant photon noise follows a Poisson distribution, the covariance matrix **Z** of the first three statistical moments of the DTOF takes the following expression [27]:

(1) $$Z=\left(\begin{array}{ccc} \text{cov}(\Delta A,\Delta A) & \text{cov}(\Delta A,\Delta T) & \text{cov}(\Delta A,\Delta V)\\ \text{cov}(\Delta T,\Delta A) & \text{cov}(\Delta T,\Delta T) & \text{cov}(\Delta T,\Delta V)\\ \text{cov}(\Delta V,\Delta A) & \text{cov}(\Delta V,\Delta T) & \text{cov}(\Delta V,\Delta V) \end{array}\right)=\left(\begin{array}{ccc} 2\frac{1}{N_{\text{tot}}} & 0 & 0\\ 0 & 2\frac{V}{N_{\text{tot}}} & 2\frac{m_{3}^{c}}{N_{\text{tot}}}\\ 0 & 2\frac{m_{3}^{c}}{N_{\text{tot}}} & 2\frac{m_{4}^{c}-V^{2}}{N_{\text{tot}}} \end{array}\right)$$

$m_{3}^{c}$, and $m_{4}^{c}$ represent the third and the fourth centralized moments of DTOF curve. The zeros in the covariance matrix indicate statistically independent measurands and non-zero terms indicate a statistical dependence. We used Monte Carlo simulations to generate data (DTOFs and sensitivity factors) for a three-layered adult human head model (scalp, skull, brain). We assume that a change in chromophores concentration can occur in two layers only: scalp and brain. The matrix **X** represents the sensitivity factors for the two layers:

(2) $$X=\left(\begin{array}{cc} {\text{MPP}}_{\text{scalp}} & {\text{MPP}}_{\text{brain}}\\ {\text{MTSF}}_{\text{scalp}} & {\text{MTSF}}_{\text{brain}}\\ {\text{VSF}}_{\text{scalp}} & {\text{VSF}}_{\text{brain}} \end{array}\right)$$

Square roots of the diagonal elements of the following covariance matrix represent the standard deviations of the estimated absorption changes in the scalp and brain layers ($\sigma \Delta \mu_{a}$) [27]:

(3) $$\sigma \Delta \mu_{a}=\sqrt{\text{diag}(\text{cov}(\Delta \mu_{a},\Delta \mu_{a}))}=\sqrt{\text{diag}({\left(X^{T}Z^{-1}X\right)}^{-1})}$$

The square root operation is carried out element-wise for the diagonal matrix.

### 2.2 Error propagation for the Beer-Lambert law

**Fig. 1.**
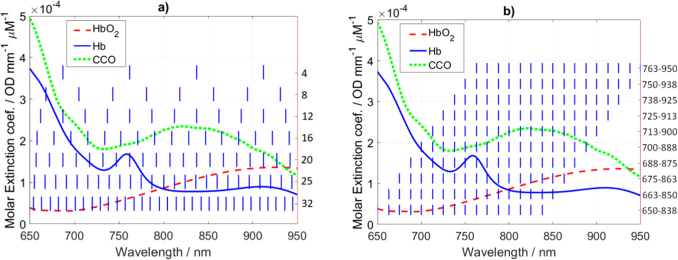
Specific extinction coefficients of oxy- (HbO_2_), deoxyhemoglobin (Hb) and cytochrome-c-oxidase (CCO) [1]. The vertical blue lines show the choices of wavelengths for the analysis of the standard deviations in the estimation of changes in chromophores concentrations for different number of wavelengths (a) and 16 consecutive wavelengths separated by 12.5 nm (b).

We determined the standard deviations in estimation of the chromophores’ concentrations: oxy- $\left(\sigma \Delta C_{\text{Hb}O_{2}}\right)$, deoxyhemoglobin ($\sigma \Delta C_{\text{Hb}})$ and cytochrome-c-oxidase ($\sigma \Delta C_{\text{CCO}}$). The implemented error propagation is similar to the method presented in [28] where authors derived the uncertainties of $\Delta C_{\text{Hb}O_{2}}$ and $\Delta C_{\text{Hb}}$ for different choices of two wavelengths. The proposed method relies on the known absorption spectra of chromophores (Fig. 1

), the calculated $\sigma \Delta \mu_{a}$ in Eq. (3), and the error propagation for the Beer-Lambert law. It was previously shown that CCO occurs in high concentration predominantly in the brain and that it is reasonable to neglect changes in CCO in the scalp for fNIRS studies [6,38,39]. Therefore, in our analysis we included twthe following mannero chromophores (oxy- and deoxyhemoglobin) in the scalp and three chromophores (including CCO) in the brain. Figure 1(a) shows 4, 8, 12, 16, 20, 25 and 32 wavelengths that evenly span the spectral range from 650 to 950 nm. Figure 1(b) shows different choices of 16 consecutive wavelengths separated by 12.5 nm.

### 2.3 Monte Carlo simulations

Twenty-five sets of DTOFs and sensitivity factor matrices (**X**) were generated using Monte Carlo simulations for wavelengths from 650 to 950 nm in steps of 12.5 nm. The Monte Carlo code was presented and explained elsewhere [37].

There were simulated $5\cdot 10^{8}$ photon packets to generate each DTOF and $10^{8}$ to generate each set of sensitivity factors. The DTOFs were sampled at *i*_max_ = 128 time channels 19.5 picoseconds each. Usually, the signal to noise ratio of a DTOF is set within the limit of 1% of the DTOF maximum and the ≥ 1% region is used for the statistical moments calculation [25]. The source-detector separation was set to 3 cm and the detector radius was set to 3 mm. The symmetry of the slab-based layered model (12×12×8 cm) allows positioning many detectors around a centrally-positioned source to significantly increase number of detected photons, hence decreasing the simulations time. Therefore, the detector was modeled as a ring of inner radius of 2.85 cm and outer radius of 3.15 cm with the center positioned at the centrally-located source. The simulations were carried out in reflectance geometry.

We assumed a three-layered model consisting of: scalp (4 mm), skull (7 mm) and brain (‘semi-infinite’). The Monte Carlo simulations generate the sensitivity factors for every layer of the modeled medium [37]. We assumed the optical properties of the skull layer remain constant as in [20,40].

### 2.4 Spectra of absorption, reduced scattering and extinction coefficients

The absorption coefficients for the scalp and the brain layers were estimated assuming tissue constituents concentrations as in [33] and using their known absorption spectra. The constituents that contribute to the absorption spectra are summarized in Table 1

**Table 1.** Tissue constituents assumed for the three layers head model [33].

Layer	Thickness (mm)	$C_{\text{Hb}O_{2}}$ (µM)	$C_{\text{Hb}}$ (µM)	$\text{St}O_{2}$ (%)	Lipid (%)	Water (%)	3 types of Cytochrome (µM)			Constant background ($mm^{-1}$)	$\mu_{a}$ ($mm^{-1}$)	
							Cytochrome c oxidase	Cytochrome b	Cytochrome c		690 nm	830 nm
Scalp	4	46	19	71	13	60	0	0	0	0.005	0.014	0.015
Skull	7	-	-	-	-	-	-	-	-	0.015	0.015	0.015
Brain	87	56	24	70	11.6	80	8	2.37	1.36	0	0.011	0.014

**Fig. 2.**
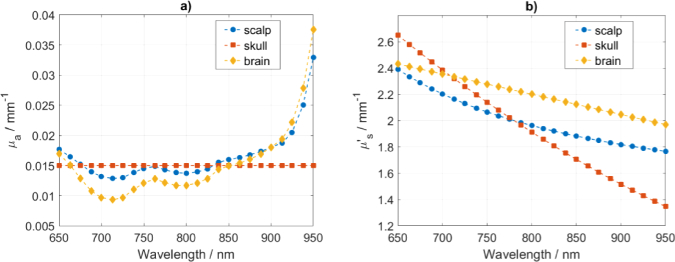
Absorption coefficient *μ*_a_ (a) and reduced scattering coefficient *μ*′_s_ (b) for scalp, skull and brain layers.

. We added a fixed background value of 0.005 mm^-1^ to the absorption of scalp making the average absorption around 0.015 mm^-1^, which is closer to values reported in [41,42]. The values of absorption assumed for the brain are around 0.013 mm^-1^, which is close to the values reported in [43–47]. The absorption of skull depends less on wavelength [48–50] and the reported values of optical properties from different studies cover a wide range [48–54]. Only minor ripples [48] can be observed in the absorption spectra of skull in the range up to 900 nm and it is suggested that these can be neglected when analyzing light penetration [55]. Thus, it was assumed that the absorption coefficient of skull is constant across all wavelengths. Moreover, the extinction coefficients of water and lipids are much smaller than of oxy-, deoxyhemoglobin and CCO in the NIR region [1]. The resulting absorption coefficients, shown in Fig. 2(a)

, are between 0.01 and 0.02 mm^-1^ for wavelengths up to 913 nm and follow values as in [56–62].

A dominant Mie regime scattering within tissues shows a decrease in the reduced scattering coefficient with wavelength (*λ*) [41–43,45,46,48,50,63]. Mie scattering can be parameterized with scattering amplitude (*a*) and power (*b*) in the following manner: *μ*′_s_ = *aλ*^-*b*^. Values of the reduced scattering coefficient in near-infrared (NIR) region are around 2 mm^-1^ for scalp [41,48], 1.8 mm^-1^ for skull [50,63] and 2.2 mm^-1^ for brain [43,47,48,64]. As such, we assumed scatter values as in [33] and in Fig. 2(b). The refractive index was set 1.4 for the media and 1 for the surrounding air.

### 2.5 Detection system: spectral responsivity (spectral efficiency)

**Fig. 3.**
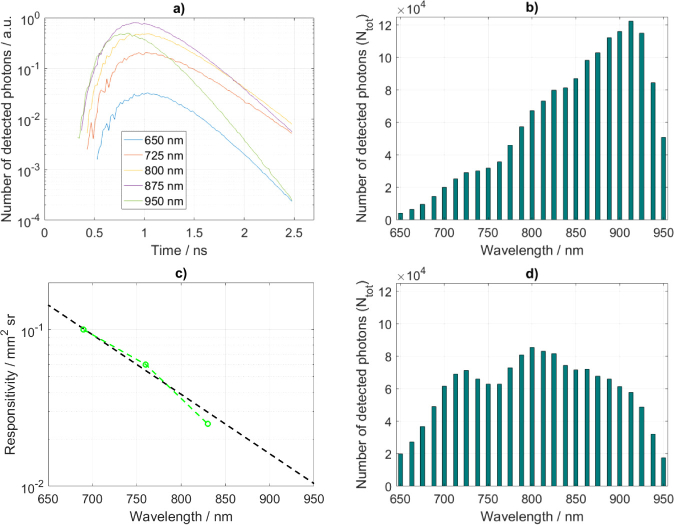
(a) Examples of the DTOFs generated for the background optical properties that are shown in Fig. 2. Number of detected photons for the case when responsivity is not used (b). The responsivity of a detector based on a multialkali cathode (c) as interpolated using data (green points) reported in [66]. Number of detected photons when the detector’s spectral responsivity is considered (d).

It is necessary to define number of detected photons ($N_{\text{tot}}$) for the calculation of the standard deviation ($\sigma \Delta \mu_{a}$ in Eq. 3). To convert the simulated photon fluence rate expressed in photons per square millimeter per second into number of photons as detected by a measurement system, simulated DTOFs are scaled to set the integral across time and wavelength to 1.5 million detected photons. The simulated DTOFs and the calculated $N_{\text{tot}}$ are shown in Fig. 3(a,b,d)

. The increase in the number of detected photons at longer wavelengths is in agreement with other studies [65]. A real detector’s sensitivity is wavelength dependent and affects the measurements as shown in [66]. We consider the spectral responsivity based on a multialkali cathode detector, which is commonly used in TR-NIRS systems [23,66]. The responsivity characteristic was interpolated from values reported in [66] and shown in Fig. 3(c). The integrals of simulated DTOFs were scaled to match the spectral responsivity characteristic and, as previously, normalized across time and wavelength to set the total detected photons to 1.5 million (Fig. 3(d)).

### 2.6 Wavelength selection analysis

**Fig. 4.**
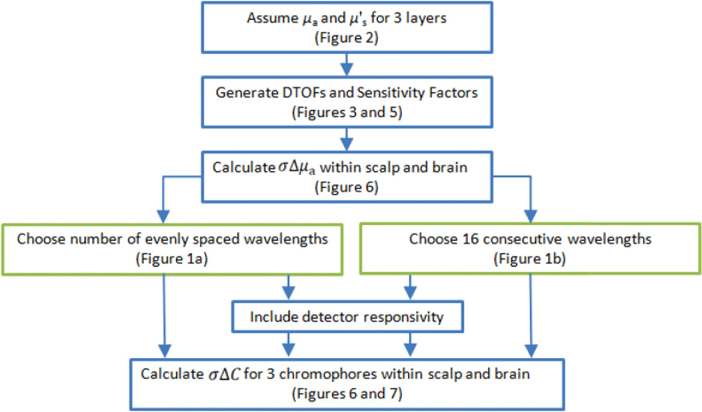
Workflow of the procedure for the calculation of standard deviations of the estimated changes in concentrations of chromophores (σΔC) using the error propagation for the moments method [27] and the Beer-Lambert law aiming to optimize wavelength choice.

The workflow of the analysis performed in this study and the steps involved are illustrated in Fig. 4

.

## 3. Results

### 3.1 Sensitivity Factors and the standard deviation of changes in absorption: $\sigma \Delta \mu_{a}$

**Fig. 5.**
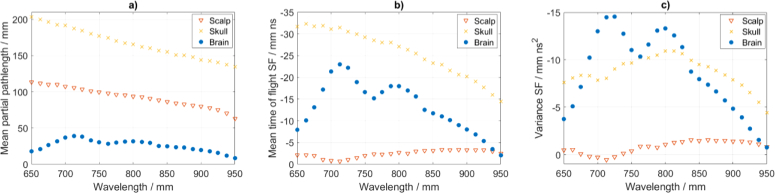
Sensitivity factors for the background optical properties as in Table 1. *MPP* (a), *MTSF* (b) and *VSF* (c) are shown within following layers: scalp, skull and brain.

DTOFs generated with the Monte Carlo simulations are shown in Fig. 3(a). MPP is expressed in units of length and represents mean distance travelled by photons passing a layer (volume). The MPP is highest within the skull layer and reduces by about a factor of two going to the scalp layer and by a further factor of two going to the brain layer (Fig. 5(a)

). On average, photons travel two times longer distance within scalp than brain. The average travel distance within brain is more than four times shorter as compared to the skull. The MTSF however is the distance travelled (MPP) weighted by the time of travel. Therefore, the maximum of MTSF is shifted to deeper layers as compared to the MPP. The MTSF is low within the scalp as the corresponding time of flight is short and increases significantly within the skull and brain (Fig. 5(b)). The VSF expresses the travelled distance (MPP) weighted by the time of travel squared, where weighting by the square of travel time pushes the maximum sensitivity even deeper. As a result, the sensitivity is further increased within the brain layer (Fig. 5(c)). Therefore, measurements of mean time of flight and variance are better suited for brain activity recovery. Furthermore, differences in the depth profiles of the MPP, MTSF and VSF support depth discrimination in parameters recovery using the moments method.

The MPP, MTSF and VSF as calculated within the scalp, skull and brain layers are shown in Fig. 5(a-c). We find that the MPP decreases with wavelength following the decrease in the values of the reduced scattering coefficient (Fig. 2(b)). The MTSF and VSF for the brain layer reveal stronger relation with the absorption than scatter as they follow the absorption spectra shape. The MPP, MTSF and VSF for the brain layer have peak values in the range between 720 and 800 nm, which follows the regions of lowest absorption coefficient as shown in Fig. 2(a).

**Fig. 6.**
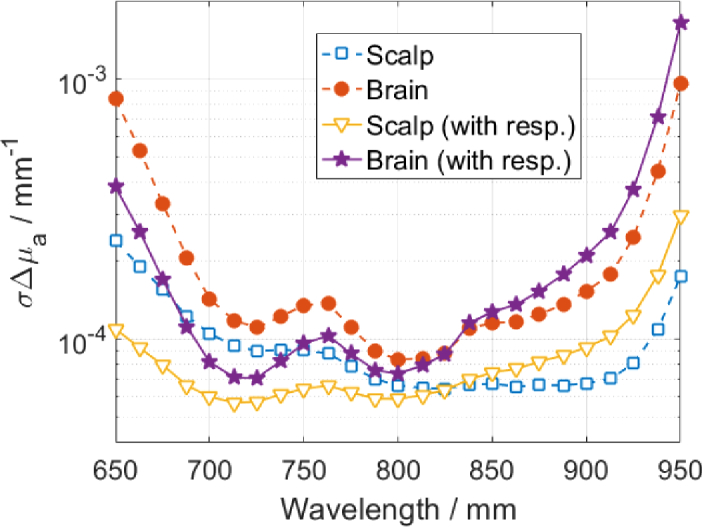
Standard deviation of changes in the absorption coefficients within two layers: scalp and brain, considering the detector responsivity (with resp.) accordingly.

The DTOFs and the sensitivity factors were used to calculate the standard deviations of changes in the absorption coefficients within the two layers (scalp and brain) using the method as introduced in section 2.1. Analyses follow methodology as in Fig. 4 and the detector spectral responsivity is considered accordingly. The calculated standard deviations at each wavelength are shown in Fig. 6

. The detector responsivity (Fig. 3(c)) introduces increase in standard deviations with wavelength, which strictly follows the detector performance. The standard deviation is always higher in the brain. Photons travel path through the scalp and skull layers is much longer than through the brain (Fig. 5(a)), hence DTOFs are dominated by information originating in the scalp and/or skull layers.

### 3.2 Varying number of wavelengths

**Fig. 7.**
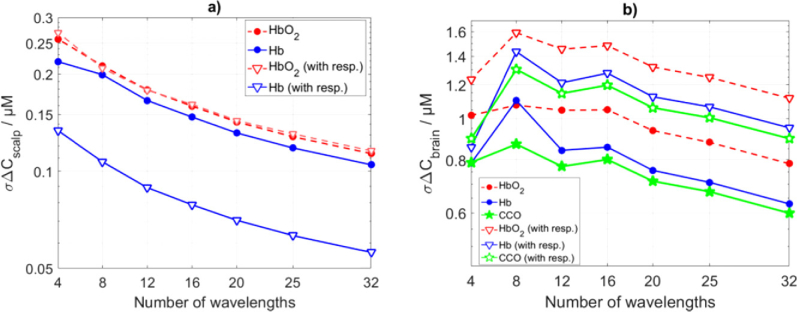
Standard deviations of estimation of changes in concentrations (σΔC) of chromophores within (a) scalp and (b) brain layers using varying number of wavelengths (as shown in Fig. 1(a)).

The standard deviations of the estimation of changes in concentrations (σΔC) of three chromophores in two layers were calculated using: standard deviations of changes in absorption coefficient $\left(\sigma \Delta \mu_{a}\right)$ as shown in Fig. 6, extinction coefficients as in Fig. 1 and the Beer-Lambert law. For the varying number of wavelengths analysis, we used MATLAB built-in function (spline) to interpolate $\sigma \Delta \mu_{a}$ in Fig. 6 for missing wavelengths. Results are presented in Fig. 7

. The standard deviation of the estimation of CCO in the brain layer $\left(\sigma \Delta C_{\text{CCO}}^{\text{brain}}\right)$ shows peak at 8 wavelengths which follows from the high values of extinction coefficients (Fig. 1(a)) and low values of $\sigma \Delta \mu_{a}$ (Fig. 6). Responsivity of the detector amplifies the standard deviation in all considered tissue constituents.

### 3.3 Varying range of 16 consecutive wavelengths

**Fig. 8.**
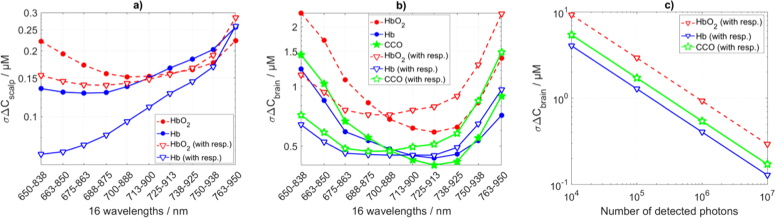
Standard deviations of estimation of changes in concentrations (σΔC) of chromophores within scalp (a) and brain (b) layers using varying range of 16 consecutive wavelengths (as shown in Fig. 2(b)). The standard deviation in change of CCO concentration in the brain at a given number of collected photons (c) for the 16 wavelengths between 700 and 888 nm. ‘with resp.’ indicates analysis considering the detector spectral responsivity.

The standard deviations of the estimated changes in concentrations (σΔC) of three chromophores in two layers using different range of 16 consecutive wavelengths separated by 12.5 nm are presented in Fig. 8(a-b)

. Choice of wavelengths region influences the σΔC within scalp and brain layers differently following difference in $\sigma \Delta \mu_{a}$ within the scalp and brain (Fig. 6) and presence of two and three chromophores in the scalp and brain respectively. The σΔC in the brain is always higher as photons travel shorter distances within the brain. Therefore, σΔC in the brain is more sensitive to the choice of wavelengths. We aim to find the wavelengths band that corresponds to minimal $\sigma \Delta C_{\text{CCO}}$ in the brain layer. In case the detector spectral responsivity is not included in the analysis, the minimal $\sigma \Delta C_{\text{CCO}}^{\text{brain}}$ of 0.40 µM is observed in the range from 725 to 913 nm (Fig. 8(b)). This region covers the absorption peak of CCO. When the detector responsivity is considered, the minimal $\sigma \Delta C_{\text{CCO}}^{\text{brain}}$ of 0.47 µM is observed in the range from 688 to 875 nm. Shorter wavelengths benefit more from the high detector responsivity than from the CCO absorption spectra. We repeated the analysis considering varying number of all detected photons $N_{\text{tot}}$. This way we can predict what is the detectable change of CCO concentration for a given number of collected photons. This analysis for the wavelength range of 700 to 888 nm is shown in Fig. 8(c). The maximum expected change in the concentration of CCO during functional brain activation is 4.5-5.5 µM [1]. The typical count rate for a spectral TR-NIRS system [25] is about $10^{6}$ photons per second.

## 4. Discussion

The error propagation analysis for the moments method was used in [27] to study how absorption coefficient recovery within two layers is influenced by the following: varying combination of statistical moments used, combinations of source-detector pairs, thickness of the top layer and source-detector distance. In this study we introduce the error propagation method to calculate the standard deviations in recovery of changes in concentrations of tissue constituents (σΔC) within head layers. We generated spectral TR-NIRS data and applied the error propagation method to optimize the choice of 16 consecutive wavelengths for the developed TR-NIRS system [25]. The optimization criteria was to minimize the uncertainty of recovery CCO concentration ($\sigma \Delta C_{\text{CCO}}$) within the brain layer. We observed comparable σΔC for the three chromophores (HbO_2_, Hb, CCO) analyzed. However, monitoring of the CCO is challenging as its low concentration requires high signal to noise ratio in raw data to detect changes in its oxidation state [1]. A recent study shows that monitoring CCO is possible with TR-NIRS [67]. Spectral responsivities vary between systems [34,67,68] and should be included in analyses. Here, we show system-specific methodology of wavelength selection as based on spectrally-resolved TR-NIRS shown in [25]. When the system responsivity was not used, the minimal $\sigma \Delta C_{\text{CCO}}$ of 0.40 µM in the brain was observed for 16 consecutive wavelengths from 725 to 913 nm and separated by 12.5 nm. This region corresponds to the broad absorption peak of CCO in the NIR region. This choice of wavelengths corresponds to systems with flat responsivity spectra, e.g. [66,67]. Inclusion of detection responsivity spectrum shifts the optimal region towards shorter wavelengths from 688 to 875 nm (minimal $\sigma \Delta C_{\text{CCO}}$ of 0.47 µM). This shift follows the detector’s spectral performance that drops with wavelength. It was recently presented that the optimal number of 3, 4, 5 and 8 wavelengths are positioned almost evenly around the peak of the absorption spectra of CCO [34]. Similarly, a broadband NIRS system presented in [69] aiming to monitor changes in CCO was set to utilize the wavelengths range between 770 and 906 nm covering the absorption peak of CCO. Almost identical range (780 to 900 nm) was used in a previous broadband NIRS system for monitoring CCO changes as presented in [14]. The recently presented TR-NIRS system used wavelengths between 780 and 870 nm when demonstrating the system’s ability to estimate changes in oxygenation and oxidate state of CCO [67]. These wavelengths correspond to the region where the performance of our detector is worse and the results of this study suggest that these wavelengths are not optimal for our system.

When changes in concentrations of multiple chromophores are of interest, a cross-talk between recovered chromophores concentrations can be expected [70]. As shown in [30,70], considering the CCO might introduce a noticeable cross-talk. However, as shown in [70] analyzing higher moments of the DTOF (e.g. the time of flight) reduces the cross-talk significantly.

The moments method [19] assumes that the probability of photons being absorbed within a layer is small, i.e. the layer absorption coefficient weighted by the mean pathlength is much smaller than 1: $\text{MPP}\cdot \Delta \mu_{a}\ll 1$. The *MPP* is derived from diffusion theory with the perturbation approach that linearize the light propagation inverse problem around some base *μ*_a_ [71] assuming the Δ*μ*_a_→0. As such, if the Δ*μ*_a_ is expected to be high as related to the base value, an iterative approach can be utilized where the sensitivity factors are iteratively recalculated using previous step recovery as the new base. This approach follows other optical tomography recovery methods [72,73]. It is the suggested next research step.

## 5. Conclusions

We proposed a method based on the error propagation allowing to calculate uncertainty of estimation of changes in concentrations of chromophores in two layers for a multi-wavelength time-resolved near-infrared spectroscopy system.

The minimal standard deviation of estimated changes in concentration of CCO within the brain layer ($\sigma \Delta C_{\text{CCO}}^{\text{brain}}$ = 0.40 µM) covered by scalp and skull was found for a set of wavelengths that cover the absorption peak of CCO: 725 to 913 nm.

For a realistic responsivitity spectrum of a system the found optimal choice of wavelengths was shifted to direction of better performance: $\sigma \Delta C_{\text{CCO}}^{\text{brain}}$ = 0.47 µM at 688 to 875 nm. The results suggest that the optimal choice of wavelengths is dependent on the specifications of a system.

## References

[1] Bale G. Elwell C. E. Tachtsidis I., “From Jobsis to the present day: a review of clinical near-infrared spectroscopy measurements of cerebral cytochrome-c-oxidase,” J. Biomed. Opt. 21(9), 091307 (2016). 10.1117/1.JBO.21.9.091307 27170072

[2] Ferrari M. Quaresima V., “A brief review on the history of human functional near-infrared spectroscopy (fNIRS) development and fields of application,” NeuroImage 63(2), 921–935 (2012). 10.1016/j.neuroimage.2012.03.049 22510258

[3] Scholkmann F. Kleiser S. Metz A. J. Zimmermann R. Mata Pavia J. Wolf U. Wolf M., “A review on continuous wave functional near-infrared spectroscopy and imaging instrumentation and methodology,” NeuroImage 85, 6–27 (2014). 10.1016/j.neuroimage.2013.05.004 23684868

[4] Modi H. N. Singh H. Yang G. Z. Darzi A. Leff D. R., “A decade of imaging surgeons’ brain function (part I): Terminology, techniques, and clinical translation,” Surgery 162(5), 1121–1130 (2017). 10.1016/j.surg.2017.05.021 28807409

[5] Pinti P. Tachtsidis I. Hamilton A. Hirsch J. Aichelburg C. Gilbert S. Burgess P. W., “The present and future use of functional near-infrared spectroscopy (fNIRS) for cognitive neuroscience,” Ann. N. Y. Acad. Sci., (2018). 10.1111/nyas.13948 PMC6367070 30085354

[6] Kolyva C. Ghosh A. Tachtsidis I. Highton D. Smith M. Elwell C. E., “Dependence on NIRS Source-Detector Spacing of Cytochrome c Oxidase Response to Hypoxia and Hypercapnia in the Adult Brain,” Adv. Exp. Med. Biol. 789, 353–359 (2013). 10.1007/978-1-4614-7411-1_47 23852515 PMC4037984

[7] Siddiqui M. F. Lloyd-Fox S. Kaynezhad P. Tachtsidis I. Johnson M. H. Elwell C. E., “Non-invasive measurement of a metabolic marker of infant brain function,” Sci. Rep. 7(1), 1330 (2017). 10.1038/s41598-017-01394-z 28465584 PMC5430960

[8] Bale G. Mitra S. de Roever I. Sokolska M. Price D. Bainbridge A. Gunny R. Uria-Avellanal C. Kendall G. S. Meek J. Robertson N. J. Tachtsidis I., “Oxygen dependency of mitochondrial metabolism indicates outcome of newborn brain injury,” J. Cereb. Blood Flow Metab. (2018). 10.1177/0271678X18777928 PMC6775592 29775114

[9] Caldwell M. Scholkmann F. Wolf U. Wolf M. Elwell C. Tachtsidis I., “Modelling confounding effects from extracerebral contamination and systemic factors on functional near-infrared spectroscopy,” NeuroImage 143, 91–105 (2016). 10.1016/j.neuroimage.2016.08.058 27591921 PMC5139986

[10] Scholkmann F. Hafner T. Metz A. J. Wolf M. Wolf U., “Effect of short-term colored-light exposure on cerebral hemodynamics and oxygenation, and systemic physiological activity,” Neurophotonics 4(04), 1 (2017). 10.1117/1.NPh.4.4.045005 PMC5695650 29181427

[11] Takahashi T. Takikawa Y. Kawagoe R. Shibuya S. Iwano T. Kitazawa S., “Influence of skin blood flow on near-infrared spectroscopy signals measured on the forehead during a verbal fluency task,” NeuroImage 57(3), 991–1002 (2011). 10.1016/j.neuroimage.2011.05.012 21600294

[12] Kirilina E. Jelzow A. Heine A. Niessing M. Wabnitz H. Bruhl R. Ittermann B. Jacobs A. M. Tachtsidis I., “The physiological origin of task-evoked systemic artefacts in functional near infrared spectroscopy,” NeuroImage 61(1), 70–81 (2012). 10.1016/j.neuroimage.2012.02.074 22426347 PMC3348501

[13] Gregg N. M. White B. R. Zeff B. W. Berger A. J. Culver J. P., “Brain specificity of diffuse optical imaging: improvements from superficial signal regression and tomography,” Front. Neuroenerg. 2, 14 (2010). 10.3389/fnene.2010.00014 PMC2914577 20725524

[14] Kolyva C. Tachtsidis I. Ghosh A. Moroz T. Cooper C. E. Smith M. Elwell C. E., “Systematic investigation of changes in oxidized cerebral cytochrome c oxidase concentration during frontal lobe activation in healthy adults,” Biomed. Opt. Express 3(10), 2550–2566 (2012). 10.1364/BOE.3.002550 23082295 PMC3469997

[15] Tachtsidis I. Leung T. S. Chopra A. Koh P. H. Reid C. B. Elwell C. E., “False positives in functional near-infrared topography,” Adv. Exp. Med. Biol. 645, 307–314 (2009). 10.1007/978-0-387-85998-9_46 19227487

[16] Tachtsidis I. Scholkmann F., “False positives and false negatives in functional near-infrared spectroscopy: issues, challenges, and the way forward,” Neurophotonics 3(3), 031405 (2016). 10.1117/1.NPh.3.3.031405 27054143 PMC4791590

[17] Eggebrecht A. T. Ferradal S. L. Robichaux-Viehoever A. Hassanpour M. S. Dehghani H. Snyder A. Z. Hershey T. Culver J. P., “Mapping distributed brain function and networks with diffuse optical tomography,” Nat. Photonics 8(6), 448–454 (2014). 10.1038/nphoton.2014.107 25083161 PMC4114252

[18] Torricelli A. Contini D. Pifferi A. Caffini M. Re R. Zucchelli L. Spinelli L., “Time domain functional NIRS imaging for human brain mapping,” NeuroImage 85(Pt 1), 28–50 (2014). 10.1016/j.neuroimage.2013.05.106 23747285

[19] Liebert A. Wabnitz H. Steinbrink J. Obrig H. Moller M. Macdonald R. Villringer A. Rinneberg H., “Time-resolved multidistance near-infrared spectroscopy of the adult head: intracerebral and extracerebral absorption changes from moments of distribution of times of flight of photons,” Appl. Opt. 43(15), 3037–3047 (2004). 10.1364/AO.43.003037 15176190

[20] Jelzow A. Wabnitz H. Tachtsidis I. Kirilina E. Bruhl R. Macdonald R., “Separation of superficial and cerebral hemodynamics using a single distance time-domain NIRS measurement,” Biomed. Opt. Express 5(5), 1465–1482 (2014). 10.1364/BOE.5.001465 24877009 PMC4026903

[21] Wabnitz H. Moeller M. Liebert A. Obrig H. Steinbrink J. Macdonald R., “Time-resolved near-infrared spectroscopy and imaging of the adult human brain,” Adv. Exp. Med. Biol. 662, 143–148 (2010). 10.1007/978-1-4419-1241-1_20 20204784

[22] Kacprzak M. Liebert A. Staszkiewicz W. Gabrusiewicz A. Sawosz P. Madycki G. Maniewski R., “Application of a time-resolved optical brain imager for monitoring cerebral oxygenation during carotid surgery,” J. Biomed. Opt. 17(1), 016002 (2012). 10.1117/1.JBO.17.1.016002 22352652

[23] Gerega A. Milej D. Weigl W. Botwicz M. Zolek N. Kacprzak M. Wierzejski W. Toczylowska B. Mayzner-Zawadzka E. Maniewski R. Liebert A., “Multiwavelength time-resolved detection of fluorescence during the inflow of indocyanine green into the adult's brain,” J. Biomed. Opt. 17(8), 087001 (2012). 10.1117/1.JBO.17.8.087001 23224200

[24] Abdalmalak A. Milej D. Norton L. Debicki D. B. Gofton T. Diop M. Owen A. M. St Lawrence K., “Single-session communication with a locked-in patient by functional near-infrared spectroscopy,” Neurophotonics 4(04), 1 (2017). 10.1117/1.NPh.4.4.040501 PMC5741990 29296627

[25] Gerega A. Milej D. Weigl W. Kacprzak M. Liebert A., “Multiwavelength time-resolved near-infrared spectroscopy of the adult head: assessment of intracerebral and extracerebral absorption changes,” Biomed. Opt. Express 9(7), 2974–2993 (2018). 10.1364/BOE.9.002974 29984079 PMC6033559

[26] Weigl W. Milej D. Gerega A. Toczylowska B. Kacprzak M. Sawosz P. Botwicz M. Maniewski R. Mayzner-Zawadzka E. Liebert A., “Assessment of cerebral perfusion in post-traumatic brain injury patients with the use of ICG-bolus tracking method,” NeuroImage 85(Pt 1), 555–565 (2014). 10.1016/j.neuroimage.2013.06.065 23831529

[27] Liebert A. Wabnitz H. Elster C., “Determination of absorption changes from moments of distributions of times of flight of photons: optimization of measurement conditions for a two-layered tissue model,” J. Biomed. Opt. 17(5), 057005 (2012). 10.1117/1.JBO.17.5.057005 22612144

[28] Yamashita Y. Maki A. Koizumi H., “Wavelength dependence of the precision of noninvasive optical measurement of oxy-, deoxy-, and total-hemoglobin concentration,” Med. Phys. 28(6), 1108–1114 (2001). 10.1118/1.1373401 11439480

[29] Corlu A. Choe R. Durduran T. Lee K. Schweiger M. Arridge S. R. Hillman E. M. C. Yodh A. G., “Diffuse optical tomography with spectral constraints and wavelength optimization,” Appl. Opt. 44(11), 2082 (2005). 10.1364/AO.44.002082 15835357

[30] Uludaǧ K. Steinbrink J. Villringer A. Obrig H., “Separability and cross talk: Optimizing dual wavelength combinations for near-infrared spectroscopy of the adult head,” NeuroImage 22(2), 583–589 (2004). 10.1016/j.neuroimage.2004.02.023 15193586

[31] Funane T. Atsumori H. Sato H. Kiguchi M. Maki A., “Relationship between wavelength combination and signal-to-noise ratio in measuring hemoglobin concentrations using visible or near-infrared light,” Opt. Rev. 16(4), 442–448 (2009). 10.1007/s10043-009-0084-6

[32] Boas D. A. Dale A. M. Franceschini M. A., “Diffuse optical imaging of brain activation: Approaches to optimizing image sensitivity, resolution, and accuracy,” NeuroImage 23, S275–S288 (2004). 10.1016/j.neuroimage.2004.07.011 15501097

[33] Correia T. Gibson A. Hebden J., “Identification of the optimal wavelengths for optical topography: a photon measurement density function analysis,” J. Biomed. Opt. 15(5), 056002 (2010). 10.1117/1.3484747 21054096

[34] Arifler D. Zhu T. Madaan S. Tachtsidis I., “Optimal wavelength combinations for near-infrared spectroscopic monitoring of changes in brain tissue hemoglobin and cytochrome c oxidase concentrations,” Biomed. Opt. Express 6(3), 933 (2015). 10.1364/BOE.6.000933 25798316 PMC4361446

[35] Re R. Contini D. Zucchelli L. Torricelli A. Spinelli L., “Effect of a thin superficial layer on the estimate of hemodynamic changes in a two-layer medium by time domain NIRS,” Biomed. Opt. Express 7(2), 264–278 (2016). 10.1364/BOE.7.000264 26977338 PMC4771447

[36] Becker W. Hickl, *16 Channel TCSPC / FLIM Detectors* (User Handbook, 1 2016).

[37] Liebert A. Wabnitz H. Żołek N. Macdonald R., “Monte Carlo algorithm for efficient simulation of time-resolved fluorescence in layered turbid media,” Opt. Express 16(17), 13188–13202 (2008). 10.1364/OE.16.013188 18711557

[38] Kolyva C. Ghosh A. Tachtsidis I. Highton D. Cooper C. E. Smith M. Elwell C. E., “Cytochrome c oxidase response to changes in cerebral oxygen delivery in the adult brain shows higher brain-specificity than haemoglobin,” NeuroImage 85(Pt 1), 234–244 (2014). 10.1016/j.neuroimage.2013.05.070 23707584 PMC3898943

[39] de Roever I. Bale G. Cooper R. J. Tachtsidis I., “Functional NIRS Measurement of Cytochrome-C-Oxidase Demonstrates a More Brain-Specific Marker of Frontal Lobe Activation Compared to the Haemoglobins,” Adv. Exp. Med. Biol. 977, 141–147 (2017). 10.1007/978-3-319-55231-6_19 28685438 PMC6126217

[40] Kacprzak M. Liebert A. Sawosz P. Zolek N. Maniewski R., “Time-resolved optical imager for assessment of cerebral oxygenation,” J. Biomed. Opt. 12(3), 034019 (2007). 10.1117/1.2743964 17614727

[41] Simpson C. R. Kohl M. Essenpreis M. Cope M., “Near-infrared optical properties of ex vivo human skin and subcutaneous tissues measured using the Monte Carlo inversion technique,” Phys. Med. Biol. 43(9), 2465–2478 (1998). 10.1088/0031-9155/43/9/003 9755939

[42] Bashkatov A. N. Genina E. A. Kochubey V. I. Tuchin V. V., “Optical properties of human skin, subcutaneous and mucous tissues in the wavelength range from 400 to 2000 nm,” J. Phys. D: Appl. Phys. 38(15), 2543–2555 (2005). 10.1088/0022-3727/38/15/004

[43] van der Zee P., “Measurement and modelling of the optical properties of human tissue in the near-infrared,” Ph.D. thesis, University College London (University of London), (1992).

[44] Bevilacqua F. Piguet D. Marquet P. Gross J. D. Jakubowski D. Venugopalan V. Tromberg B. J. Depeursinge C., “Superficial tissue optical property determination using spatially resolved measurements close to the source: Comparison with Frequency Domain Photon Migration measurements,” Proc. SPIE 3597, 540–547 (1999). 10.1117/12.356857

[45] Bevilacqua F. Piguet D. Marquet P. Gross J. D. Tromberg B. J. Depeursinge C., “In vivo local determination of tissue optical properties: applications to human brain,” Appl. Opt. 38(22), 4939 (1999). 10.1364/AO.38.004939 18323984

[46] Yaroslavsky A. N. Schulze P. C. Yaroslavsky I. V. Schober R. Ulrich F. Schwarzmaier H. J., “Optical properties of selected native and coagulated human brain tissues in vitro in the visible and near infrared spectral range,” Phys. Med. Biol. 47(12), 2059–2073 (2002). 10.1088/0031-9155/47/12/305 12118601

[47] Zee P. V. D. Essenpreis M., “Optical properties of brain tissue,” Proc. SPIE 1888, 454–465 (1993). 10.1117/12.154665

[48] Firbank M. Hiraoka M. Essenpreis M. Delpy D. T., “Measurement of the optical properties of the skull in the wavelength range 650-950 nm,” Phys. Med. Biol. 38(4), 503–510 (1993). 10.1088/0031-9155/38/4/002 8488176

[49] Ugryumova N. Matcher S. J. Attenburrow D. P., “Measurement of bone mineral density via light scattering,” Phys. Med. Biol. 49(3), 469–483 (2004). 10.1088/0031-9155/49/3/009 15012014

[50] Bashkatov A. N. Genina E. A. Kochubey V. I. Tuchin V. V., “Optical properties of human cranial bone in the spectral range from 800 to 2000nm,” in Saratov Fall Meeting 2005 (SPIE 2006), p. 11.

[51] Farzam P. Lindner C. Weigel U. Suarez M. Urbano-Ispizua A. Durduran T., “Noninvasive characterization of the healthy human manubrium using diffuse optical spectroscopies,” Physiol. Meas. 35(7), 1469–1491 (2014). 10.1088/0967-3334/35/7/1469 24901605

[52] Farzam P. Zirak P. Binzoni T. Durduran T., “Pulsatile and steady-state hemodynamics of the human patella bone by diffuse optical spectroscopy,” Physiol. Meas. 34(8), 839–857 (2013). 10.1088/0967-3334/34/8/839 23859825

[53] Pifferi A. Torricelli A. Taroni P. Bassi A. L. Chikoidze E. Giambattistelli E. Cubeddu R., “Optical biopsy of bone tissue: a step toward the diagnosis of bone pathologies,” J. Biomed. Opt. 9(3), 474–480 (2004). 10.1117/1.1691029 15189084

[54] Xu Y. Iftimia N. Jiang H. Key L. Bolster M., “Imaging of in vitro and in vivo bones and joints with continuous-wave diffuse optical tomography,” Opt. Express 8(7), 447–451 (2001). 10.1364/OE.8.000447 19417840

[55] Jacques S. L., “Optical properties of biological tissues: a review,” Phys. Med. Biol. 58(11), R37–R61 (2013). 10.1088/0031-9155/58/11/R37 23666068

[56] Custo A. Wells W. M. 3rd Barnett A. H. Hillman E. M. Boas D. A., “Effective scattering coefficient of the cerebral spinal fluid in adult head models for diffuse optical imaging,” Appl. Opt. 45(19), 4747–4755 (2006). 10.1364/AO.45.004747 16799690

[57] Montcel B. Chabrier R. Poulet P., “Time-resolved absorption and hemoglobin concentration difference maps: a method to retrieve depth-related information on cerebral hemodynamics,” Opt. Express 14(25), 12271–12287 (2006). 10.1364/OE.14.012271 19529655

[58] Comelli D. Bassi A. Pifferi A. Taroni P. Torricelli A. Cubeddu R. Martelli F. Zaccanti G., “In vivo time-resolved reflectance spectroscopy of the human forehead,” Appl. Opt. 46(10), 1717–1725 (2007). 10.1364/AO.46.001717 17356614

[59] Barnett A. H. Culver J. P. Sorensen A. G. Dale A. Boas D. A., “Robust inference of baseline optical properties of the human head with three-dimensional segmentation from magnetic resonance imaging,” Appl. Opt. 42(16), 3095–3108 (2003). 10.1364/AO.42.003095 12790461

[60] Sawosz P. Wojtkiewicz S. Kacprzak M. Weigl W. Borowska-Solonynko A. Krajewski P. Bejm K. Milej D. Ciszek B. Maniewski R. Liebert A., “Human skull translucency: post mortem studies,” Biomed. Opt. Express 7(12), 5010–5020 (2016). 10.1364/BOE.7.005010 28018721 PMC5175548

[61] Jager M. Kienle A., “Non-invasive determination of the absorption coefficient of the brain from time-resolved reflectance using a neural network,” Phys. Med. Biol. 56(11), N139–N144 (2011). 10.1088/0031-9155/56/11/N02 21572234

[62] Okada E. Delpy D. T., “Near-infrared light propagation in an adult head model. I. Modeling of low-level scattering in the cerebrospinal fluid layer,” Appl. Opt. 42(16), 2906–2914 (2003). 10.1364/AO.42.002906 12790439

[63] Torricelli A. Pifferi A. Taroni P. Giambattistelli E. Cubeddu R., “In vivo optical characterization of human tissues from 610 to 1010 nm by time-resolved reflectance spectroscopy,” Phys. Med. Biol. 46(8), 2227–2237 (2001). 10.1088/0031-9155/46/8/313 11512621

[64] Azimipour M. Baumgartner R. Liu Y. Jacques S. L. Eliceiri K. Pashaie R., “Extraction of optical properties and prediction of light distribution in rat brain tissue,” J. Biomed. Opt. 19(7), 075001 (2014). 10.1117/1.JBO.19.7.075001 24996660

[65] Sato H. Kiguchi M. Kawaguchi F. Maki A., “Practicality of wavelength selection to improve signal-to-noise ratio in near-infrared spectroscopy,” NeuroImage 21(4), 1554–1562 (2004). 10.1016/j.neuroimage.2003.12.017 15050579

[66] Wabnitz H. Taubert D. R. Mazurenka M. Steinkellner O. Jelzow A. Macdonald R. Milej D. Sawosz P. Kacprzak M. Liebert A. Cooper R. Hebden J. Pifferi A. Farina A. Bargigia I. Contini D. Caffini M. Zucchelli L. Spinelli L. Cubeddu R. Torricelli A., “Performance assessment of time-domain optical brain imagers, part 1: basic instrumental performance protocol,” J. Biomed. Opt. 19(8), 086010 (2014). 10.1117/1.JBO.19.8.086010 25121479

[67] Lange F. Dunne L. Hale L. Tachtsidis I., “MAESTROS: A Multiwavelength Time-Domain NIRS System to Monitor Changes in Oxygenation and Oxidation State of Cytochrome-C-Oxidase,” IEEE J. Sel. Top. Quantum Electron. 25(1), 1–12 (2019). 10.1109/JSTQE.2018.2833205 PMC6054019 30450021

[68] Re R. Contini D. Turola M. Spinelli L. Zucchelli L. Caffini M. Cubeddu R. Torricelli A., “Multi-channel medical device for time domain functional near infrared spectroscopy based on wavelength space multiplexing,” Biomed. Opt. Express 4(10), 2231–2246 (2013). 10.1364/BOE.4.002231 24156079 PMC3799681

[69] Bale G. Mitra S. Meek J. Robertson N. Tachtsidis I., “A new broadband near-infrared spectroscopy system for in-vivo measurements of cerebral cytochrome-c-oxidase changes in neonatal brain injury,” Biomed. Opt. Express 5(10), 3450–3466 (2014). 10.1364/BOE.5.003450 25360364 PMC4206316

[70] Uludag K. Kohl M. Steinbrink J. Obrig H. Villringer A., “Cross talk in the Lambert-Beer calculation for near-infrared wavelengths estimated by Monte Carlo simulations,” J. Biomed. Opt. 7(1), 51 (2002). 10.1117/1.1427048 11818012

[71] Arridge S. R., “Photon-measurement density functions. Part I : Analytical forms,” Appl. Opt. 34(31), 7395–7409 (1995). 10.1364/AO.34.007395 21060614

[72] Arridge S. R., “Optical tomography in medical imaging,” Inverse Problems 15(2), R41–R93 (1999). 10.1088/0266-5611/15/2/022

[73] Dehghani H. Eames M. E. Yalavarthy P. K. Davis S. C. Srinivasan S. Carpenter C. M. Pogue B. W. Paulsen K. D., “Near infrared optical tomography using NIRFAST: Algorithm for numerical model and image reconstruction,” Commun. Numer. Methods Eng. 25(6), 711–732 (2009). 10.1002/cnm.1162 PMC2826796 20182646

